# Wearable Microfluidic Sensor for the Simultaneous and Continuous Monitoring of Local Sweat Rates and Electrolyte Concentrations

**DOI:** 10.3390/mi13040575

**Published:** 2022-04-06

**Authors:** Yuki Hashimoto, Takako Ishihara, Kei Kuwabara, Tatsuro Amano, Hiroyoshi Togo

**Affiliations:** 1 NTT Device Innovation Center, NTT Corporation, 3-1, Atsugi 243-0198, Japan; takako.ishihara.ty@hco.ntt.co.jp (T.I.); kei.kuwabara.kn@hco.ntt.co.jp (K.K.); hiroyoshi.togo.wp@hco.ntt.co.jp (H.T.); 2 Laboratory for Exercise and Environmental Physiology, Faculty of Education, Niigata University, Niigata 950-2181, Japan; amano@ed.niigata-u.ac.jp

**Keywords:** sensor, wearable, sweat, flow rate, electrolyte concentration

## Abstract

Temperature elevation due to global warming increases the risks of dehydration, which can induce heat-related illness. Proper rehydration with appropriate amounts of water and electrolytes is essential to aid body fluid homeostasis. Wearable sweat sensors which can monitor both the sweat rate and sweat electrolyte concentration may be an effective tool for determining appropriate rehydration. Here, we developed a novel potentially wearable sensor that can monitor both the local sweat rate and sweat electrolyte concentration continuously. The new device includes a system with a short microfluidic pathway that guides the sweat appearing on the skin to a small space in the device to form a quantifiable droplet. The sweat rate is assessed from the time for the droplet to appear and droplet volume, while an integrated electric sensor detects the sodium chloride concentration in each sweat droplet. We demonstrated that this new device could record both the flow rates of artificial sweat and its sodium chloride concentration in ranges of human sweating with an accuracy within ±10%. This is equivalent to the accuracy of commercially available sweat rate meters and sweat ion sensors. The present study provides a new perspective for the design of wearable sensors that can continuously monitor sweat rates and sweat electrolyte concentrations for potential application to a healthcare device.

## 1. Introduction

Global warming is increasing the incidence of heat waves [1,2] and heat-related illness [3] around the world. Sweating is a vital physiological function to cool the human body during heat stress [4]. However, excessive sweating without rehydration induces body fluid and electrolytes (e.g., sodium) loss, which disturbs human body homeostasis and potentially causes heat-related illness. Appropriate rehydration to counter the fluid and electrolyte loss is essential for preventing heat-related illness during physical activity in hot environments.

The volume of fluid loss through sweating can be easily assessed by measuring body weight reduction after heat stress. However, assessing sweat electrolyte loss requires sensors attached to skin. To calculate the electrolyte loss, a sensor must be able to measure both the local sweat volume and the electrolyte concentration. Physiological studies have shown that the sweat sodium concentration varies widely across a range of sweat production [5] in which the response may differ among individuals [6]. This indicates that sodium loss cannot be properly interpreted from the assessment of sweat sodium concentration alone. Another important factor for sweat analysis is continuous measurement. Studies have shown that sweat electrolyte concentration may increase rapidly at a certain production of sweat [7,8]. Continuous measurement of both the sweat rate and sweat sodium concentration may detect the initiation of rapid sodium loss through sweating during heat stress. This feedback may help in establishing a better rehydration strategy during exercise or heat exposure in hot conditions.

Sweat absorbent patches, the whole-body-washdown technique, and polymer bags/films have been used for evaluating sweat rates and sweat compositions during heat stress [9,10]. However, these methods require the use of professional equipment, involve proper protocol for sample storage [11], lack real-time monitoring capability, carry a potential risk of sweat sample evaporation and contamination [10], and are inefficient in terms of cost, all of which may limit their broader utility. Emerging studies have proposed wearable sweat sensors that eliminate the procedure (and thus time) for sweat collection and analysis, allowing the continuous monitoring of sweat without professional experts and equipment [12]. A number of wearable sweat sensors have been reported so far [12]. Representative methods for quantifying the concentration of specific components of sweat can be divided into two categories: electrochemical measurement with ion-selective electrode [13,14,15,16,17,18,19,20] and the colorimetric method [21,22,23,24].

Electrochemical measurement with an ion-selective electrode is a technique to implement electrochemical detection technology in a wearable device and continuously monitor the concentration of specific components of sweat in a form where the device is adhered to the skin. This approach is likely preferable, as it uses a small and fully integrated wearable device that can continuously monitor the concentration of a specific component of sweat through an external device such as a smartphone [13]. On the other hand, fresh sweat secreted on the skin surface mixes with old sweat. This presents a challenge for the device because the sensor readings are not in real time, but show a rolling average of the analysis profile—the device has no way to control the flow of sweat so that only fresh sweat is detected.

The colorimetric method quantifies the concentration of a specific component in sweat by coloring the sweat in microfluidic channels using colorimetric reactions [21]. This method is attractive because, as opposed to the electrochemical measurement method, it does not require the integration of a power supply circuit, analog–digital signal processing circuit, and wireless communications circuit into the device. Furthermore, the structure comprises only a flow channel and colorimetric material without batteries, and it only requires an easily accessible external optical detection device such as a smartphone. The fluidic system guides old sweat to controlled micro channels via sweat secretion pressure and capillary action, allowing the chemical analysis of fresh sweat appearing on the skin without any artifact contamination. Although this system can quantify sweat rates [23], the measurement principle of the colorimetric method makes it unsuitable for monitoring sweat volume and electrolyte concentration continuously. On the other hand, the fluidic system itself has been utilized for the detection of biomarkers [25,26], and is considered an effective tool in this case.

To overcome the problems in continuously measuring rates of fresh sweat and its chemical components, we developed an original sensor principle and verified its outcome. Our new device based on this principle includes a system with a short microfluidic pathway that guides sweat appearing on the skin to a small chamber to form a droplet—a quantifiable form—to assess sweat volume. It also includes an electric sensor to detect the sodium chloride concentration in each sweat droplet, allowing the continuous and simultaneous monitoring of both the sweat volume and sweat sodium chloride concentration.

## 2. Materials and Methods

### 2.1. Sensing Method

**Figure 1:**
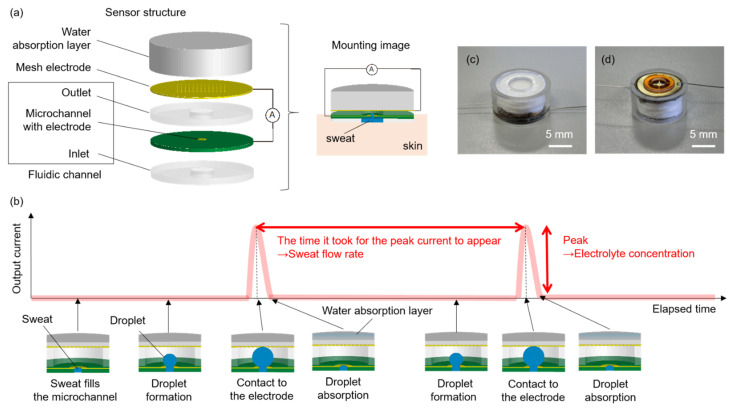
(**a**) Schematic illustration of the sensor mounted on skin. (**b**) Sensing protocol for sweat flow rate and electrolyte concentration monitoring. The graph schematically shows the time-dependent fluctuation of the output current obtained from the sensor output along with illustrations of droplet formation in the sensor chamber corresponding to the changes in output currents. (**c**) Fabricated sensor (top view). (**d**) Fabricated sensor (bottom view).

As shown in Figure 1a, the sensor is mainly composed of a fluidic channel, two electrodes, and a water absorption layer to mount the device on human skin. The fluidic channel includes a microchannel between the inlet and outlet layers. The outlet layer is hydrophobic. When actually attaching the sensor to the skin, a belt-type jig such as a wristband, double-sided tape, or adhesive reagent is used to ensure sufficient adhesion between the inlet layer surface and the skin. One electrode is placed between the inlet layer and microchannel. The other electrode has a mesh structure and is placed between the outlet and water absorption layers. Figure 1b describes the sensing procedure. Once sweat appears on the skin under the device, it fills the microchannel and subsequently forms a droplet in the space above the outlet layer. When the droplet contacts the mesh electrode, electric current is generated between the electrodes. Subsequently, the current flow quickly disappears because the droplet is captured by the water absorption layer, due to capillary action. The appearance of current conduction due to the droplet’s formation and its disappearance are repeated according to the provision of sweat. The sweat flow rate can be estimated from the droplet volume and the time to generate the droplet. The electrolyte concentration can be estimated from the peak value in the obtained current. The distance between the two electrodes is an important design parameter and defines the upper limit of the droplet volume that can be formed between the electrodes. For example, considering the case where this sensor is attached to the human body, the distance should be so small that the effect of inertial forces caused by body motions is negligible. Specifically, the distance should be designed so that the Eötvös number, which is a dimensionless number measuring the importance of inertial forces compared to surface tension forces, is less than 1. The time during which the current is flowing, i.e., from the time the droplet contacts the mesh electrode until it is collected in the water absorption layer through the mesh electrode, is also an important parameter. Since the time determines the upper limit of the sweat flow rate that can be detected by the sensor, it is necessary to shorten the time to obtain a higher detection limit. On the other hand, if the time is too short, high sampling rate for the measurement is required.

### 2.2. Experimental Setup

A prototype device (Figure 1c,d) was fabricated to demonstrate the validity. A polytetrafluoroethylene substrate with a gold-plated through-hole (diameter of 0.30 mm; height of 0.25 mm) was used as the microchannel with the electrode. A polydimethylsiloxane substrate with a hole (diameter of 3.0 mm; height of 0.50 mm) fabricated by conventional soft lithography with photo-curable polydimethylsiloxane (KER-4690-A/B, Shin-Etsu Chemical Co., Ltd., Tokyo, Japan) [27,28,29,30] and polycarbonate substrate with a hole (diameter of 4.0 mm; height of 0.50 mm) were used as the inlet and outlet, respectively. Silver fabric sheet with submillimeter-scale lattice-like structures (AGposs, Mitsufuji, Japan, diameter of 10.0 mm) and polypropylene blended pulp (diameter of 10.0 mm; thickness of 5.0 mm) were used as the mesh electrode and water absorption layer, respectively. The height of the outlet (0.5 mm) corresponds to the distance between the two electrodes. The calculated Eötvös number under inertial force of 1 G was around 0.008. This value enables droplet formation in which surface tension forces are dominant to inertial forces caused by body motions in the wearable measurement. Saline solution (range, 1.0–200 mM) was injected into the sensor with a syringe pump (PUMP 11 ELITE, Harvard Apparatus, Holliston, MA, USA) directly connected to the fluidic channel as a model replicating sweat appearance. The output current was monitored with a digital multimeter (34465A, Keysight Technologies, Santa Rosa, CA, USA). In the measurement, the flow channel in the sensor was rinsed once with ultrapure water before the next measurement at a different NaCl concentration, in order to eliminate the influence of the previous concentrations. We confirmed that the droplet was collected in the water absorption layer through the mesh electrode in about 30 ms, long enough to easily detect the peak current at a moderate sampling rate. In addition, the sensor was confirmed to be able to repeatedly measure for at least 6 h at a constant flow rate (1.0 µL/(min·cm^2^)) whose value corresponded to standard local sweat flow rate in exercise.

### 2.3. Data Analysis

General-purpose numerical analysis software (MATLAB R2021b, MathWorks, Natick, MA, USA) was used to extract the time it took for the current peak to appear, and the peak values from the output current were acquired by the digital multimeter.

## 3. Results and Discussion

**Figure 2:**
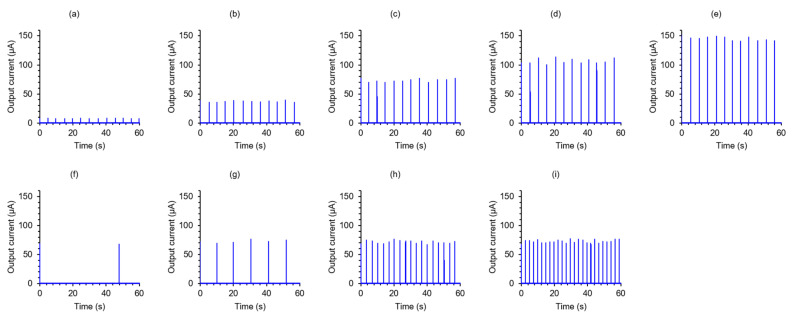
Typical changes in output current obtained from the electrochemical sensor when the sodium chloride concentration in the artificial sweat was fixed with the input flow rate changed (**a**–**e**) and vice versa (**f**–**i**). (**a**) 10 mM, 1.0 µL/min. (**b**) 50 mM, 1.0 µL/min. (**c**) 100 mM, 1.0 µL/min. (**d**) 150 mM, 1.0 µL/min. (**e**) 200 mM, 1.0 µL/min. (**f**) 100 mM, 0.1 µL/min. (**g**) 100 mM, 0.5 µL/min (**h**) 100 mM, 1.5 µL/min. (**i**) 100 mM, 2.0 µL/min.

**Figure 3:**
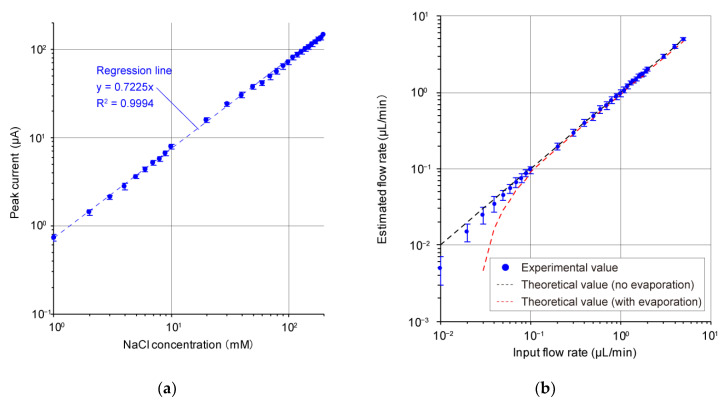
(**a**) Relationship between sodium chloride (NaCl) concentration in artificial sweat introduced into the microchannel and the obtained electrochemical peak current. (**b**) Relationship between artificial sweat flow rate in the microchannel and the estimated flow rate calculated from the recorded current signal. The data are shown as mean ± the 95% confidence interval.

Figure 2 shows the time-dependent changes in the output current obtained from the electrochemical sensor, which was sensitive to sodium chloride in ranges of 1 to 200 mM, with an artificial sweat flow rate of 0.1 to 2.0 µL/min, mimicking ranges of human sweating. When the sodium chloride concentration was increased at a fixed artificial sweat flow rate (Figure 2a–e), the peak current increased. The change is attributed to the increase in conductivity of the artificial sweat following the increase in sodium chloride concentration [31,32,33]. The time it took for the current peak to appear (i.e., the frequency of sweat drop formation) was not altered by the changes in sodium chloride concentration. Figure 3a shows the change in peak output current recorded from the artificial sweat at various concentrations of sodium chloride under the experimental conditions described above. It was observed that the peak current increased linearly with increasing sodium chloride concentration in a range of 1 to 200 mM. The robust linear relationship between the electrical conductivity of sodium chloride solution and actual sodium chloride concentration is consistent with previous studies [31,32,33]. This suggests that our device measured reasonable electric properties. We confirmed that the sodium chloride concentration in the artificial sweat could be estimated from the peak value with an error of ±10% based on the regression line obtained from the present study (Figure 3a). This is sufficient and reasonable accuracy, equivalent to that of commercially available electrochemical sensors sensitive to sodium chloride [34]. In addition, the detection limit can be estimated to be 0.18 mM from the experimental result. This value is considered to be sufficient to detect in human sweat, since the concentration range of sodium and chloride ions in human sweat is from 10 to 100 mM [35].

When the input flow rate of the artificial sweat was increased at a fixed sodium chloride concentration (Figure 2c,f–i), the time it took for the current peak to appear was also increased. The magnitude of the peak current fluctuated slightly. We think this result is due to the fact that the time required for the droplet to be absorbed by the mesh electrode and the water absorption layer is in the order of milliseconds, which is fast enough compared to the rate of droplet formation. Figure 3b shows the estimated flow rate calculated from the time it took for the peak output current (peak to peak) to appear when the artificial sweat was introduced into the microchannel at various input flow rates. The input flow rate, *Q* [L/s] was estimated using the time for the peak current to appear, *T* [s], and the maximum volume of droplet, *V_max_* [L], as follows:

(1) $$Q=\frac{V_{max}}{T}$$

Here, V was set so that the estimated value was closest to the reference value (*V_max_* = 82.5 nL). When the input flow rate was less than 0.1 µL/min, the estimated value was smaller than the reference value, as shown by the black dash line in Figure 3b, probably due to a pronounced evaporation effect. In addition, in order to analyze the effect of evaporation, the estimated values calculated by Equation (1) are shown by red dotted lines when the effect of evaporation is present. For this analysis, we used the following equations to find the time t at which the droplet volume *V*[t] reaches *V_max_* and converts it to flow rate:

(2) $$V\left[t\right]=\frac{\pi r^{3}\left(2-3\cos\theta \left[t\right]+\cos^{3}\theta \left[t\right]\right)}{\sin^{3}\theta \left[t\right]}$$

(3) $$\frac{d\theta \left[t\right]}{dt}=\frac{\sin^{4}\theta \left[t\right]}{\pi r^{3}{\left(1-\cos\theta \left[t\right]\right)}^{2}}\cdot Q_{in}-\frac{\sin^{3}\theta \left[t\right]}{\pi r^{2}\left(1-\cos\theta \left[t\right]\right)}\cdot \frac{2\pi D\left(C_{0}-C_{\infty }\right)}{\rho }$$

where *Q_in_* [L/s] is the input flow rate into the sensor, *r* [m] is the radius of the microchannel, *θ* [*t*] [rad] is the contact angle of the droplet at time *t*, *D* [m^2^/s] is the diffusion coefficient of water vapor, *C_0_* is the water vapor concentration at the air–liquid interface of the droplet, *C_∞_* is the water vapor concentration sufficiently far from the air–liquid interface of the droplet, *ρ* [kg/m^3^] is the density of water. The calculations were performed with *r* = 1.5 × 10^−4^ [m], *θ*[0] = 1 [deg], D = 2.91 × 10^−5^ [m^2^/s], *C_0_* = 0.01743 [kg/m^3^], *C_∞_* = 0.01177 [kg/m^3^], and *ρ* = 1.0 × 10^3^ [kg/m^3^], respectively. The absence of data in this graph for flow rates of less than 0.02 µL/min indicates that no droplet is produced because the evaporation rate of the droplet is larger relative to the input flow rate. Considering these results, we assume that the evaporation of the droplets is suppressed by the effect of the sodium chloride solution suspended in the sweat model solution compared to the case where the droplets in the sensor are water droplets, and thus the error of the estimated input flow rate is reduced relative to the reference value. On the other hand, for input flow rates of 0.1 µL/min or higher, i.e., in cases where sweat production is relatively large, such as sweating in hot conditions, the effect of evaporation was hardly observed, and the error of the estimated value of the input flow rate with respect to the reference value was kept within ±10% of the reading. This is a sufficient and reasonable error in comparison to commercially available sweat meters for assessing thermal sweat production [36]. In addition, the detection limit can be estimated to be 0.1 µL/min based on the experimental result. The lower limit of the local sweat flow rate in human thermal sweating is reported to be 0.1 µL/(min·cm^2^) [37], even in the case of elderly people whose sweating function has declined. Considering the above, we believe that designing the cross-sectional area of the inlet in the sensor to be 1 cm^2^ will provide sufficient measurement accuracy for the wearable measurement.

We believe that the proposed device can be used to establish a proper hydration strategy for people during exercise or heat exposure in hot climates. In addition, by developing electrodes that are more selective and specific to other molecules or ions, it could also be utilized as a healthcare device for monitoring several biomarkers in sweat such as glucose, lactate acid, and alcohol. Further study is required to develop a structure to reduce the effect of sweat evaporation in the sensor so that a small amount of sweat can be measured. Finally, a proof-of-concept study in human participants is obligatory in the future.

## 4. Conclusions

We proposed a new concept for a potentially wearable sweat sensor to continuously measure the flow rate of sweat and its electrolyte concentration. We also evaluated the accuracy of a prototype sensor and found that it was acceptable for monitoring both the sweat rate and sweat’s sodium chloride concentration in wide ranges of volume and concentration, respectively. Our results indicate that the proposed sensor is potentially viable for monitoring sweat flow rates and electrolyte concentrations in actual human sweating. They also shed light on the design of wearable sensors for monitoring sweat volume and the concentration of specific components in sweat.
